# Tibial plateau fracture morphology based on injury force mechanism is predictive for patient-reported outcome and conversion to total knee arthroplasty

**DOI:** 10.1007/s00068-024-02447-5

**Published:** 2024-01-20

**Authors:** Nick Assink, Thijs P. Vaartjes, Eelke Bosma, Sven H. van Helden, Joost G. ten Brinke, Harm Hoekstra, Frank F. A. IJpma

**Affiliations:** 1 Department of Trauma Surgery, University of Groningen, University Medical Center Groningen, HPC BA13, Hanzeplein 1, 9713 GZ Groningen, The Netherlands; 2 3D Lab, University of Groningen, University Medical Center Groningen, Groningen, The Netherlands; 3 Department of Trauma Surgery, Martini Hospital, Groningen, The Netherlands; 4 Department of Trauma Surgery, Isala Hospital, Zwolle, The Netherlands; 5 Department of Trauma Surgery, Gelre Hospital, Apeldoorn, The Netherlands; 6 Department of Traumatology, KU Leuven University Hospitals Leuven Gasthuisberg Campus, Louvain, Belgium

**Keywords:** Tibial plateau fractures, Injury force mechanism, Fracture morphology, Varus, Valgus, KOOS

## Abstract

**Purposes:**

The aim of this study was to assess the relationship between injury mechanism–based fracture patterns and patient-reported outcome as well as conversion rate to total knee arthroplasty (TKA) at follow-up.

**Methods:**

A multicenter cross-sectional study was performed including 1039 patients treated for a tibial plateau fracture between 2003 and 2019. At a mean follow-up of 5.8 ± 3.7 years, patients completed the Knee injury and Osteoarthritis Outcome Score (KOOS) questionnaire. For all patients, the injury force mechanism was defined based on CT images. Analysis of variance (ANOVA) was used to assess the relationship between different injury mechanisms and functional recovery. Cox regression was performed to assess the association with an increased risk on conversion to TKA.

**Results:**

A total of 378 (36%) patients suffered valgus-flexion, 305 (29%) valgus-extension, 122 (12%) valgus-hyperextension, 110 (11%) varus-flexion, 58 (6%) varus-hyperextension, and 66 (6%) varus-extension injuries. ANOVA showed significant different KOOS values between injury fracture patterns in all subscales (*P* < 0.01). Varus-flexion injuries had the lowest average KOOS scores (symptoms 65; pain 67; ADL 72; sport 35; QoL 48). Varus-flexion mechanism was associated with an increased risk on a TKA (HR 1.8; *P* = 0.03) whereas valgus-extension mechanism was associated with a reduced risk on a TKA (HR 0.5; *P* = 0.012) as compared to all other mechanisms.

**Conclusion:**

Tibial plateau fracture patterns based on injury force mechanisms are associated with clinical outcome. Varus-flexion injuries have a worse prognosis in terms of patient-reported outcome and conversion rate to TKA at follow-up. Valgus-extension injuries have least risk on conversion to TKA.

## Introduction

Tibial plateau fractures are one of the most challenging intra-articular fractures to treat due to complex fracture morphology [1, 2]. These fractures result from a varus or valgus load along with or without an axial load on the tibial plateau [3, 4]. Depending on the exact injury mechanism and the position of the knee, the resulting fracture patterns vary from simple split fractures to complex multi-fragmentary fractures of lateral, medial, or bicondylar types [1, 5, 6].

Both the revisited Schatzker and the three-column classification emphasized the importance of the injury mechanisms causing a tibial plateau fracture [7, 8]. Both classifications included the assessment of these mechanisms in four dimensions (varus, valgus, flexion, and extension) in order to guide surgical fixation. In addition, the hyperextension injury mechanism has been reported as a unique fracture mechanism [4, 9], which resulted in a total of six different unique tibial plateau injury mechanisms: valgus-flexion, valgus-extension, valgus-hyperextension, varus-flexion, varus-extension, and varus-hyperextension. Xie et al. recently introduced a method to assess the relationship between these different injury force mechanisms and fracture patterns [4]. This study demonstrated that those injury force mechanisms — represented by those unique fracture patterns — predict associated soft tissue injuries. Besides the descriptive nature of these mechanisms, these specific injury patterns may have predictive value on the patient’s recovery over time. Even though Xie et al. identified distinct mechanism-associated 3-dimensional pattern characteristics, these well-established mechanisms — which are incorporated in the current classification mechanisms — have never been associated with functional recovery [4].

In this study, we aim to assess the relationship between the different unique tibial plateau injury mechanisms and the functional recovery at follow-up. We posed the following research questions: (1) Is the type of injury force mechanism which causes a tibial plateau fracture predictive for patient-reported functional outcome at follow-up? (2) What is the association between the type of injury force mechanism and the risk on conversion to total knee arthroplasty (TKA) at follow-up?

## Methods

A multicenter cross-sectional study was performed including all patients who have been treated for a tibial plateau fracture in five trauma centers (University Medical Center Groningen, Martini Hospital, Isala Hospital, Gelre Hospital, and KU Leuven University Hospitals) between January 2003 and December 2019. Patients were eligible for inclusion based upon the availability of a preoperative (diagnostic) CT scan of the injured knee. Patients with a follow-up of less than 1 year, age < 18 years, pathological fractures, isolated tibial eminence fractures, or those with a complicated fracture requiring amputation of the affected leg were excluded. Patients’ demographics were retrieved from the electronic records. All patients were verified whether they were still alive according to the national population registry. All eligible patients were approached by posted mail, asked for informed consent, and asked to complete validated patient-reported outcome measures. Written informed consent was obtained from all participants.

### Fracture injury mechanism assessment

**Fig. 1:**
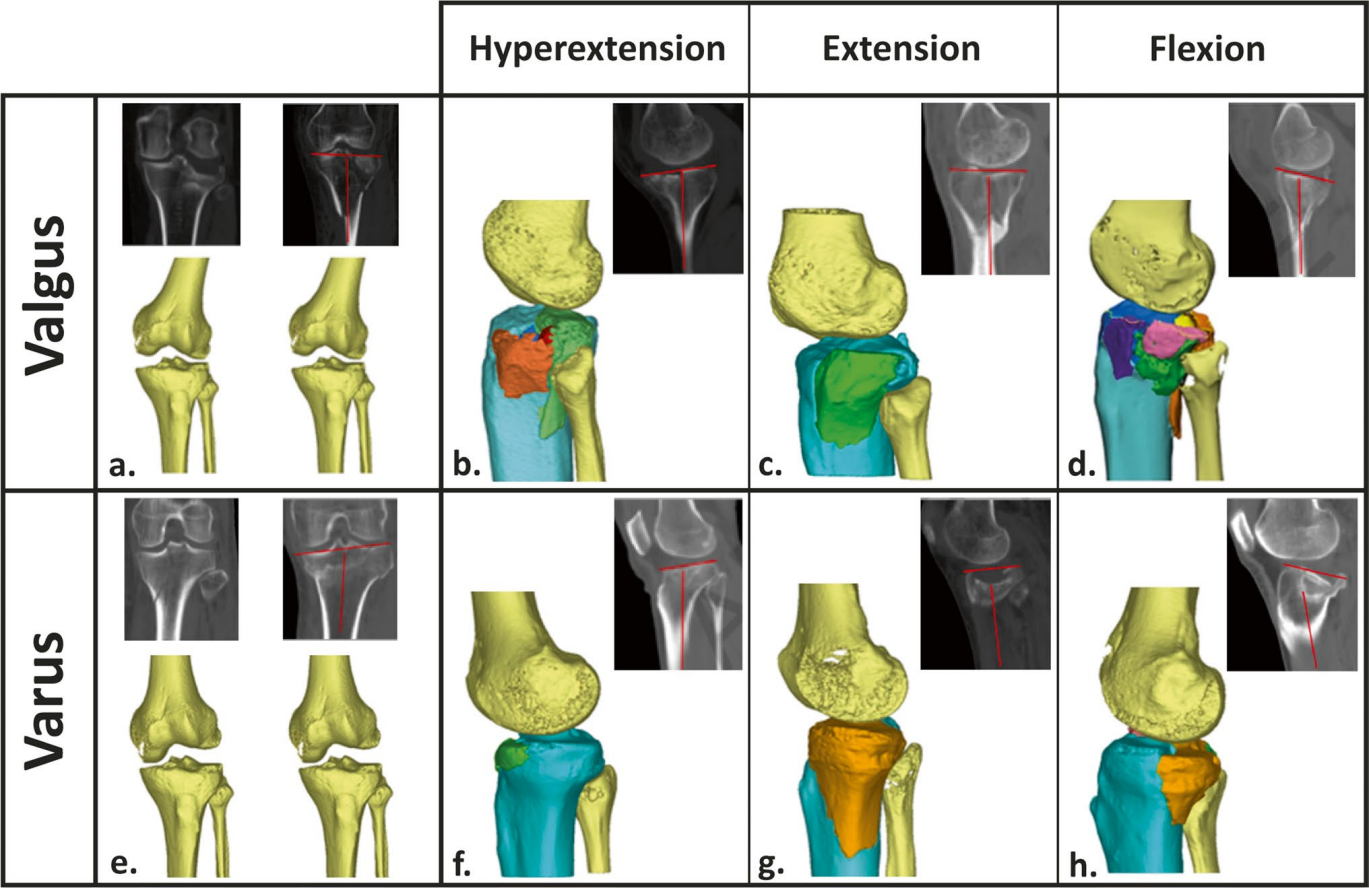
Fracture injury mechanism assessment. Fractures limiting to the lateral side or with an increased medial proximal tibial angle (MPTA) are considered caused by valgus impact (**a**) and fractures limited to the medial side or with decreased MPTA as varus impact (**e**). Fractures with a decreased tibial slope are considered as hyperextension (**b**, **f**), normal tibial slope as extension (**c**, **g**), and increased tibial slope as flexion (**d**, **h**)

Fracture injury mechanism was assessed in consensus of two independent observers. Any disagreements were solved during a consensus meeting with a third observer. Assessments were performed on the 2D CT slices using the Mimics Medical software package (Version 23.0, Materialise, Leuven, Belgium) according to the method described by Xie et al. [4]. Additionally, the injury mechanism was verified on a 3D reconstruction of the fracture. This reconstruction was obtained following a segmentation process in which a preset bone threshold (Hounsfield unit ≥ 226) was used combined with the “region growing” function in order to remove the femur bone. Figure 1 illustrates the fracture injury mechanism assessment.

### Patient-reported outcomes

All eligible patients were approached by posted mail and asked to complete the standardized Knee injury and Osteoarthritis Outcome Score (KOOS) questionnaire [10]. Additionally, patients were asked whether they received a total knee prothesis. The KOOS is a validated questionnaire consisting of five subscales: pain, symptoms, activities of daily living (ADL), function in sport and recreation (sport), and knee related quality of life (QoL). A normalized score was calculated for each subscale. Scores of the subscales are calculated by summing up the individual items (e.g., questions) and transforming scores on a range from 0 to 100, with higher scores indicating better function. In addition, the patients were also asked whether they underwent conversion to TKA.

### Statistical analysis

IBM SPSS software, version 23.0 for Windows (IBM Corporation, Chicago, IL, USA), was used for statistical analysis. Continuous variables ware presented as mean and standard deviation (SD) for normally distributed data and median and interquartile range (IQR) if not normally distributed. Descriptive statistics were used to describe the study population. The study population was divided into groups based on the injury mechanism, after which analysis of variance (ANOVA) was used to assess differences between the groups in terms of functional outcome. Cox regression was performed to assess the risk on conversion to a TKA. In this analysis, we corrected for other factors (age, sex, smoking, and BMI) which are potential confounders for the risk of conversion to a TKA. A *P*-value of less than 0.05 was considered statistically significant.

### Ethical approval

The institutional review board of all centers approved the study procedures, and the research was performed in accordance with the relevant guidelines and regulations. This study is reported following the Strengthening the Reporting of Observational Studies in Epidemiology (STROBE) reporting guideline [11].

### Source of funding

There was no external funding source for this study.

## Results

A total of 2331 patients were treated for a tibial plateau fracture between 2003 and 2019, of which 61 had and isolated tibial eminence avulsions (e.g., cruciate ligament injuries), 115 were aged < 18 years, 191 had died at follow-up, 82 had co-existing conditions complicating outcome measurement (e.g., Parkinson, paralysis), 4 had an amputation, 13 had no knowledge of the Dutch language, and 53 had an unknown address or were lost at follow-up, leaving 1750 patients eligible for follow-up analysis. All patients were approached by posted mail, from which 1039 responded (response rate 59%) at a mean follow-up of 5.8 ± 3.7 years. The mean age was 53 ± 15 years and 32% (329) of patients were male. A total of 728 (70%) patients were treated operatively by using plate and/or screw osteosynthesis. Eventually, 111 (11%) patients underwent conversion to TKA during follow-up. Non-response analysis demonstrated that non-responders were slightly younger (51 ± 18 vs. 53 ± 15, *P* = 0.001), less often female (59% vs. 68%, *P* = 0.001), and less often treated surgically (56% vs. 70%, *P* = 0.001) in comparison with responders.

Valgus mechanisms (805/1039, 77%) occurred more frequently than varus (234/1039, 23%) mechanisms. A total of 378 (36%) patients were classified as valgus-flexion, 305 (29%) as valgus-extension, 122 (12%) as valgus-hyperextension, 110 (11%) as varus-flexion, 58 (6%) as varus-hyperextension, and 66 (6%) as varus-extension. Table 1 describes patients demographics for each trauma mechanism.

**Table 1:** Patient characteristics

	Valgus-flexion	Valgus-extension	Valgus-hyperextension	Varus-flexion	Varus-extension	Varus-hyperextension
Number of patients	378 (36%)	305 (29%)	122 (12%)	110 (11%)	58 (6%)	66 (6%)
Age (yrs)	54 ± 14	54 ± 15	51 ± 15	53 ± 17	55 ± 16	46 ± 14
Male	98 (65%)	100 (33%)	46 (38%)	33 (32%)	22 (38%)	30 (45%)
BMI (kg/m^2^)	26.2 ± 4.8	26.3 ± 4.7	25.4 ± 4.0	26.5 ± 4.5	26.6 ± 4.2	27.4 ± 5.5
Smoking	73 (19%)	68 (22%)	26 (21%)	22 (20%)	8 (14%)	13 (20%)
Diabetes	28 (7%)	28 (9%)	12 (10%)	6 (6%)	7 (12%)	3 (5%)
AO/OTA classification						
B1	20 (5%)	48 (16%)	5 (4%)	10 (9%)	17 (30%)	26 (39%)
B2	109 (29%)	94 (31%)	22 (18%)	8 (7%)	9 (16%)	5 (8%)
B3	167 (44%)	148 (48%)	80 (66%)	38 (34%)	19 (32%)	15 (22%)
C1	12 (3%)	1 (0%)	6 (5%)	4 (4%)	6 (10%)	7 (11%)
C2	2 (1%)	3 (1%)	3 (2%)	2 (2%)	0 (0%)	6 (9%)
C3	68 (18%)	11 (4%)	6 (5%)	48 (44%)	7 (12%)	7 (11%)
Treatment						
Non-operative	117 (31%)	94 (31%)	14 (11%)	25 (22%)	32 (55%)	29 (44%)
Screw osteosynthesis	38 (10%)	65 (21%)	13 (11%)	2 (2%)	3 (5%)	8 (12%)
Plate osteosynthesis	223 (59%)	146 (48%)	95 (78%)	83 (76%)	23 (40%)	29 (44%)
Conversion to TKA	46 (12%)	22 (7%)	11 (9%)	20 (18%)	8 (14%)	4 (6%)
Follow-up (yrs)	5.6 ± 3.6	6.1 ± 3.9	5.6 ± 3.6	5.3 ± 3.3	5.9 ± 3.9	6.6 ± 4.2

### Patient-reported outcomes

**Fig. 2:**
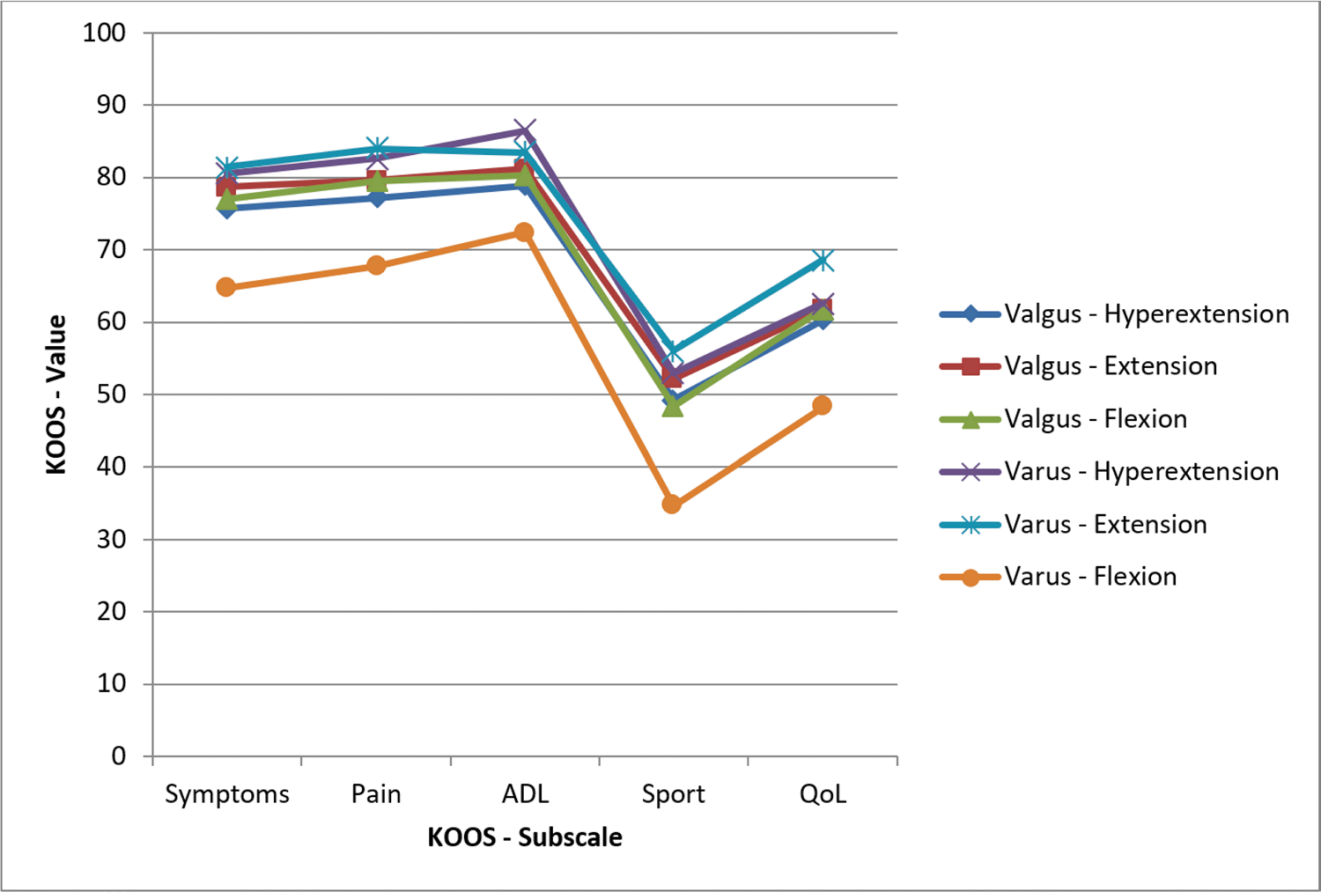
The average KOOS values for each of the KOOS subscales, representing functional outcome, are displayed for 944 patients with a tibial plateau fracture divided into six subgroups based on the injury fracture mechanism

The average KOOS score per KOOS subscale for each trauma mechanism is depicted in Fig. 2. ANOVA analysis showed significant different KOOS values between injury mechanism groups in terms of all KOOS subscales (*P* < 0.001, Appendix). Tukey’s post hoc analysis showed that patients with a fracture caused by a varus-flexion injury had significantly worse KOOS subscales regarding symptoms (*P* ≤ 0.002), pain (*P* ≤ 0.007), sport (*p* ≤ 0.01), and QoL (*P* ≤ 0.026) as compared to all other mechanisms. In terms of ADL, varus-flexion was significantly worse as compared to all other subscales with exemption of valgus-hyperextension (*P* = 0.012).

### Conversion to TKA

**Fig. 3:**
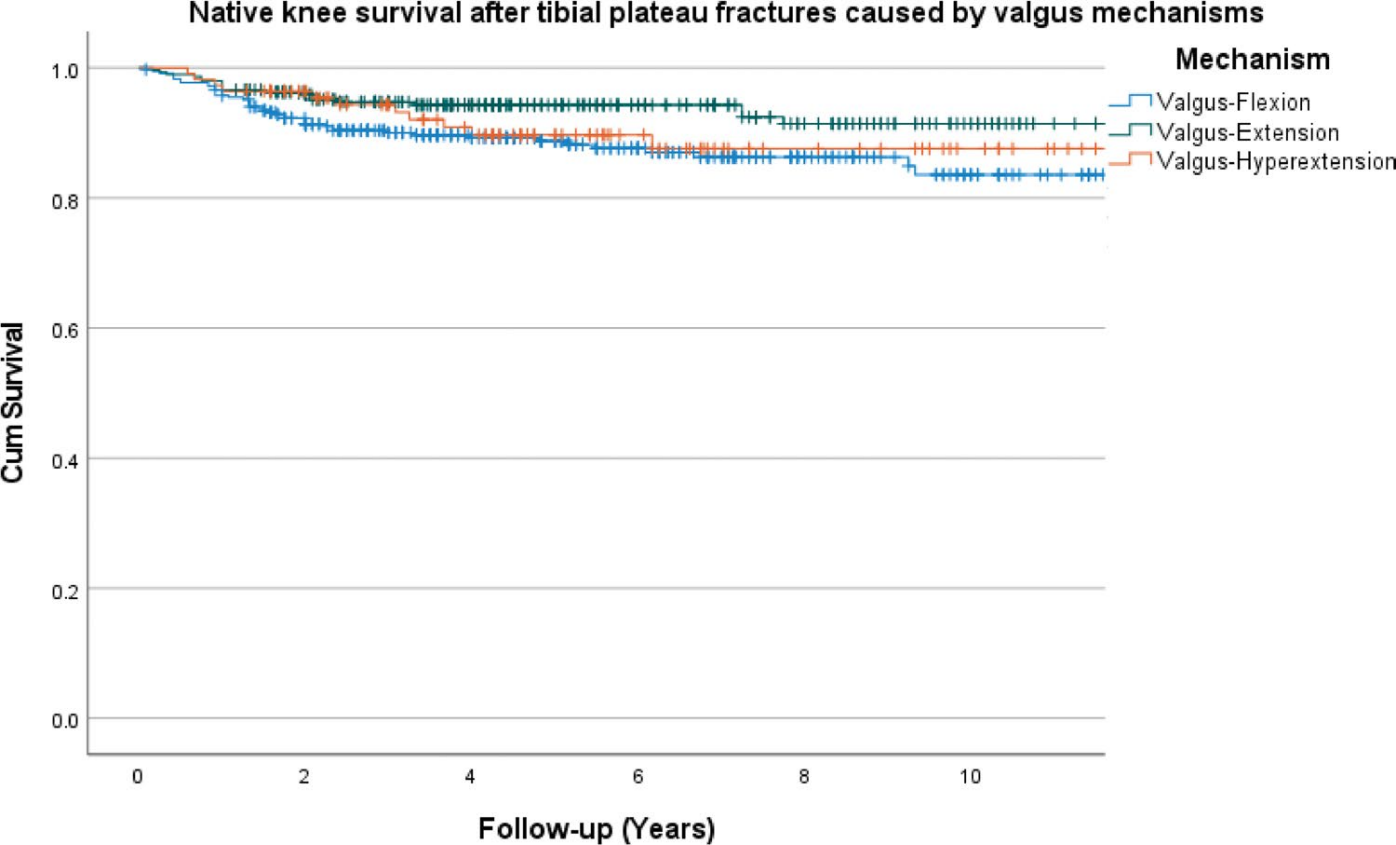
Native knee survival free from conversion to total knee arthroplasty stratified by valgus injury mechanisms (log rank, *P* = 0.010)

**Fig. 4:**
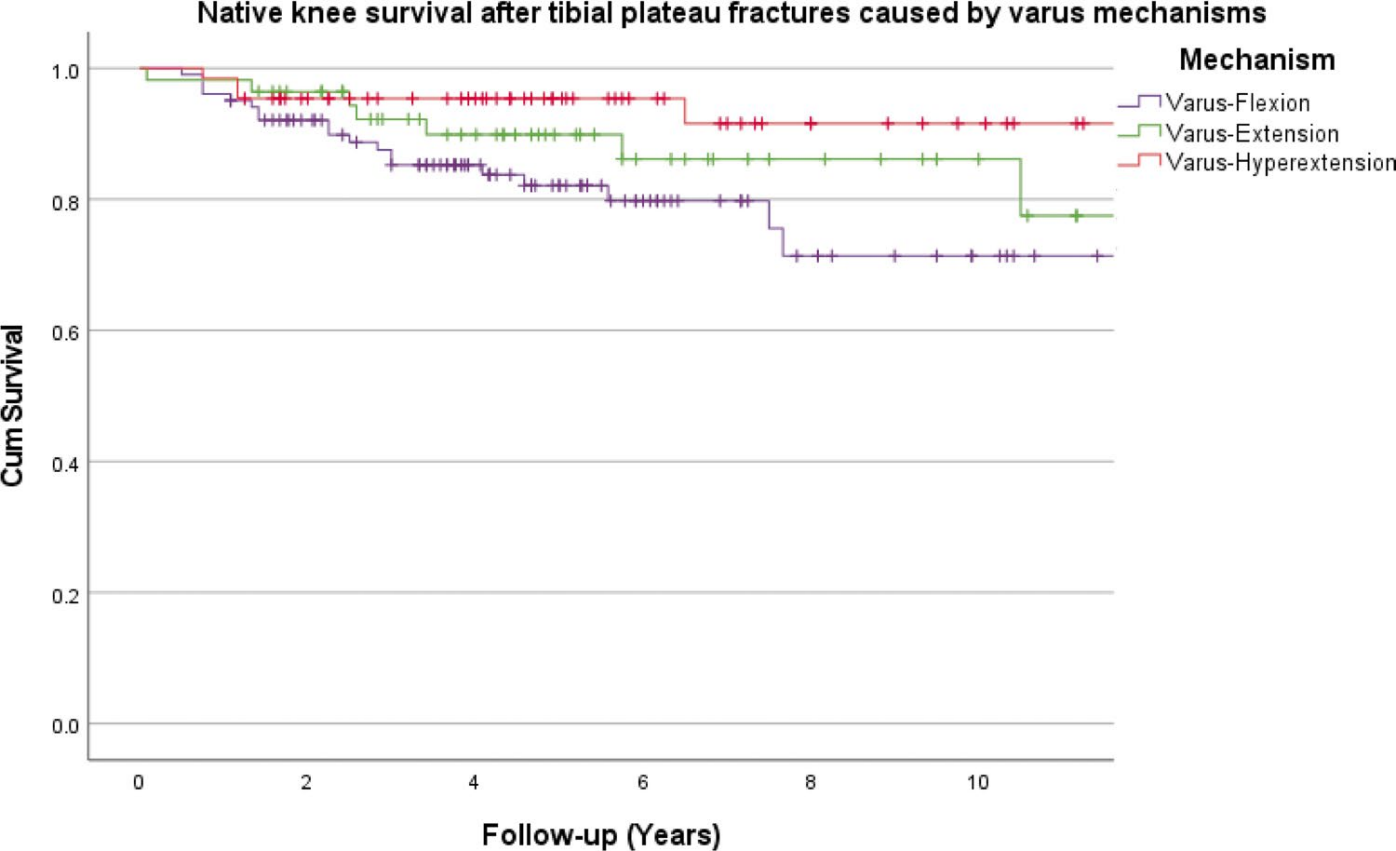
Native knee survival free from conversion to total knee arthroplasty stratified by varus injury mechanisms (log rank, *P* = 0.075)

Kaplan–Meier survival analysis shows knee survival free of conversion to TKA in non-surgical treated patients of 97% at 2 years and 94% at 10 years. In surgically treated patients, 2- and 10-year knee survival was 93% and 82%, respectively. When stratifying groups based on the injury force mechanism, the 2-year knee survival (no conversion to TKA) was 92% for valgus-flexion, 96% for valgus-extension, and 96% for valgus-hyperextension. For varus-flexion, extension, and hyperextension injuries, the 2-year knee survival was 92%, 90%, and 95%, respectively. The 10-year knee survival was 84% for valgus-flexion, 91% for valgus-extension, 88% for valgus-hyperextension, 71% for varus-flexion, 86% for varus-extension, and 92% for varus-hyperextension (Figs. 3 and 4).

Univariate analysis shows that the valgus-extension mechanism was associated with a reduced risk on a TKA (HR 0.6; *P* = 0.028), whereas the varus-flexion mechanism was associated with an increased risk on a TKA (HR 2.0; *P* = 0.003) as compared to the other mechanisms. After correction for age, sex, smoking, diabetes, and BMI, multivariate analysis showed similar results with valgus-extension mechanism associated with a reduced risk (adj HR 0.5; *P* = 0.012) and varus-flexion with increased risk on a TKA (adj HR 1.8; *P* = 0.030) (Table 2).

**Table 2:** Multivariate analysis of the association of injury force mechanisms with the conversion to TKA

Injury mechanism^+^	Unadjusted hazard ratio (95% CI)	*P*-value	Adjusted hazard ratio† (95% CI)	*P*-value
Valgus-flexion	1.2 (0.8–1.8)	0.333	1.4 (0.9–2.1)	0.105
Valgus-extension	0.6 (0.3–0.9)	0.028*	0.5 (0.3–0.9)	0.012*
Valgus-hyperextension	0.8 (0.4–1.5)	0.777	0.9 (0.5–1.7)	0.750
Varus-flexion	2.0 (1.3–3.3)	0.003*	1.8 (1.1–3.1)	0.030*
Varus-extension	1.3 (0.7–2.8)	0.428	0.8 (0.3–2.0)	0.672
Varus-hyperextension	0.6 (0.2–1.5)	0.246	0.9 (0.3–2.5)	0.847

^+^The patients who had a different injury force mechanism as the injury mechanism of interest served as the reference group

^†^Adjusted for age, sex, smoking, diabetes, and BMI

^*^Significant

### Why injury force mechanisms affect clinical outcome

A subanalysis was performed to assess the role of revision surgeries as potential explanation for the identified variations in clinical outcomes among different injury mechanisms. Varus-flexion injuries are associated with substantial rates of revision surgery as a result of fracture-related infections (13%), residual displacement (8%), or meniscal/ligamentous repairs (7%) as compared to the other injury mechanisms. Table 3 presents rates of revision surgery pertaining to different injury mechanisms in tibial plateau fracture surgery.

**Table 3:** Revision surgery associated with different injury force mechanisms in tibial plateau fracture surgery

	Valgus-flexion	Valgus-extension	Valgus-hyperextension	Varus-flexion	Varus-extension	Varus-hyperextension
Number of patients	378 (36%)	305 (29%)	122 (12%)	110 (11%)	58 (6%)	66 (6%)
Operative treatment	261 (67%)	211 (69%)	108 (89%)	85 (77%)	26 (45%)	37 (56%)
Revision surgery						
Reoperation for fracture-related infection*	15 (6%)	4 (2%)	1 (1%)	11 (13%)	0 (0%)	0 (0%)
Revision surgery for residual displacement	7 (2%)	2 (1%)	3 (3%)	9 (8%)	0 (0%)	0 (0%)
Reoperation for meniscal or ligamentous repair	8 (2%)	4 (1%)	1 (1%)	8 (7%)	1 (2%)	4 (6%)

^*^As percentage of patients treated operatively

## Discussion

Tibial plateau fracture morphologies based on six different injury force mechanisms, each with its own associated distinct fracture characteristics and soft tissue involvement, have been introduced [4]. To our knowledge, this is the first large multicenter study which relates these specific injury mechanisms to patients’ functional recovery in terms of patient-reported outcome and conversion to TKA. Our results show that especially fractures caused by a varus-flexion force have worse prognosis as compared to the other mechanisms regarding both patient-reported outcome and risk on conversion to TKA. On the contrary, fractures caused by a valgus-extension mechanism are associated with reduced risk on a TKA as compared to the other injury mechanisms.

In the recently updated versions of the Schatzker and three-column classification methods, more focus is directed on mechanisms of injury which result in different fracture patterns [7, 8]. In these classifications, identifying the position of the knee (flexion/extension) and the deforming force (varus/valgus) guides the surgeon in the preoperative planning and surgical stabilization of the fracture [7, 8]. The results of our study add knowledge regarding the consequences of these mechanisms on patients’ prognosis. Therefore, increasing knowledge about the injury mechanism and the corresponding fracture morphology could aid in providing the patient information regarding his or her expected functional recovery.

The results of this study indicate that especially the varus-flexion mechanism results in worse patient-reported outcomes as compared to other mechanisms of injury. These fractures are caused by a varus force, with the knee in a flexion position. Therefore, these fractures usually involve the medial and posterior part of the tibial plateau. Recently, van den Berg et al. showed that especially fractures with posterior involvement as well as sagittal malalignment were associated with poor outcomes [12, 13]. It is believed that fractures of the medial plateau usually require more force and are therefore often not only limited to the medial compartment. This is confirmed by Xie et al. who describe that varus-flexion fractures are associated with posterolateral articular comminution and anterior cruciate ligament avulsion [4]. The combination of a medial fracture with posterolateral comminution and associated ligamental damage in fractures caused by a varus-flexion trauma might explain the worse clinical outcome. This is supported by our subanalysis which showed that patients who had a fracture caused by a varus-flexion injury required more often reoperations for meniscal or ligamentous repair, residual displacement, and fracture-related infections as compared to other mechanisms of injury. Therefore, fractures caused by a varus-flexion trauma might especially benefit from an extensive 3D surgical planning which could potentially improve surgical outcomes [14]. Moreover, valgus traumas are associated with medial collateral ligament (MCL) sprains which are relatively forgiving, whereas varus traumas are associated with (postero)lateral ligamental injuries which are often underestimated and could affect outcome negatively [15].

Tibial plateau fractures could result in severe post-traumatic osteoarthritis or ligamentous instability needing eventually conversion to total knee arthroplasty. This study shows that patients with a fracture caused by a flexion injury mechanism are more likely to receive a TKA as compared to the other injury mechanisms. Varus-flexion mechanisms appear to be almost two times more likely to undergo conversion to a TKA compared to the other injury mechanisms (adj HR 1.8, 95% CI, 1.1–3.1). Also, the valgus-flexion mechanism showed a trend towards an increased risk of conversion to TKA (adj HR 1.4, 95% CI, 0.9–2.1). This increased risk in fractures caused by a flexion force could be explained by the involvement of the posterior part of the tibial plateau as well as the relatively high incidence of C3 fractures. Our findings are in line with recent research, indicating that inadequate alignment of the sagittal tibial axis is strongly associated with conversion to TKA [16]. On the contrary, the valgus-extension mechanism was associated with a reduced risk on conversion to a TKA compared to other groups (adj HR 0.5, 95% CI, 0.3–0.9). This reduced risk could be explained by the fact that these fractures are mainly limited to the lateral compartment of the tibial plateau and usually consist of solely a central depression and/or pure split fragment [4].

This study has a few limitations which need to be addressed. First, we acknowledge that selection bias is inherent to a cross-sectional study design caused by loss to follow-up and non-response. Non-response analysis demonstrates that non-responders were on average 2 years younger and more often female. Yet, the small difference in age and gender is not expected to affect the generalizability of our results. Interestingly, when comparing our patient group with the patients described by Xie et al., our population has a higher proportion of females and less fractures caused by a varus force. This may be due to demographic and cultural differences between western Europe and China. Second, our research solely focusses on the relationship between the injury force mechanism and patient-reported outcome as well as conversion to TKA. For future research, a more detailed comparison between injury force mechanisms regarding both the fractures’ location and initial displacement of tibial plateau fractures and of postoperative reduction based on two-dimensional CT slices and even more advanced three-dimensional imaging techniques would be helpful to further elucidate factors that affect prognosis. [17, 18]. Third, our subanalysis demonstrated some differences in reoperations due to soft tissue injuries between injury mechanisms. Although, the true level of concomitant ligamental and meniscal damage presented at the time of injury is still matter of debate. A recent review indicated that at least one ligamental or meniscal lesion is present in 93% of patients with tibial plateau fractures [19]. This again emphasizes the importance of a full assessment of concomitant soft tissue injuries in especially tibial plateau fractures with posterolateral involvement caused by varus-flexion mechanisms [20]. A preoperative MRI could therefore be useful in selected cases and contribute to decision-making regarding treatment strategies. In addition, a preoperative MRI could help to quantify the condition of the cartilage and the extent of preexisting osteoarthritis. We envision for the future to tibial plateau fracture management a three-dimensional diagnostic workup in which fracture characteristics and a full soft tissue assessment will be combined [17].

In conclusion, this large multicenter study demonstrated that tibial plateau fracture morphology based on injury force mechanism is predictive for patient-reported outcome and conversion to total knee arthroplasty. This study showed that in particular fractures caused by a varus-flexion force have a worse prognosis, whereas fractures caused by a valgus-extension force have less risk on conversion to a TKA. These findings can help in patient counselling, identifying patients who might benefit from advanced preoperative workup (i.e., MRI/3D surgical planning), and estimating prognosis in the management of complex tibial plateau fractures.

## Data Availability

Not applicable.

## Appendix

Table 4

**Table 4:** Injury mechanisms correlated with patient-reported outcome

	Valgus-flexion	Valgus-extension	Valgus-hyperextension	Varus-flexion	Varus-extension	Varus-hyperextension	*P*-value
No. of fractures	378	305	122	110	58	66	
KOOS symptoms	76.8 ± 21.5	78.2 ± 20.7	75.7 ± 22.5	65.0 ± 21.9	80.9 ± 22.1	80.8 ± 18.0	< 0.001*
KOOS pain	78.9 ± 21.9	79.1 ± 21.8	77.1 ± 22.5	67.0 ± 22.5	83.8 ± 19.4	82.9 ± 19.7	< 0.001*
KOOS ADL	80.0 ± 21.9	80.8 ± 21.2	79.1 ± 22.2	71.7 ± 22.5	83.0 ± 22.8	86.7 ± 16.8	< 0.001*
KOOS sport	48.4 ± 35.4	51.4 ± 34	50.7 ± 36.5	34.6 ± 29.1	54.5 ± 38.3	53.7 ± 32.8	< 0.001*
KOOS quality of life	61.2 ± 28.9	60.9 ± 28.8	60.3 ± 29.5	47.8 ± 24.7	68.2 ± 28.1	63.2 ± 26.1	< 0.001*

*Significant
